# Toward isolating perceptual and physiological contributors to heat sensitivity in multiple sclerosis: insights from a new experimental model

**DOI:** 10.1007/s00421-025-05838-7

**Published:** 2025-06-11

**Authors:** Timothy English, Joshua Barton, Nicole Vargas, Michael Barnett, Ollie Jay

**Affiliations:** 1 Heat and Health Research Centre, Faculty of Medicine and Health, University of Sydney, Sydney, Australia; 2 Beach Brain, Southern Cross University Hospital, Birtinya, Australia; 3 Institute for Climate, Energy and Disaster Solutions, College of Health and Medicine, Australian National University, Acton, Australia; 4 Multiple Sclerosis Clinic, Brain and Mind Centre, University of Sydney, Sydney, Australia

**Keywords:** Multiple sclerosis, Heat, Skin cooling, Symptom, Visual evoked potentials, Neural conduction

## Abstract

**Purpose:**

To determine if reductions in whole-body thermal sensation (WBTS) with localised skin cooling mitigate heat-induced visual performance decrements in people with multiple sclerosis (MS), optic neuritis, and heat-sensitive visual symptoms, independent of core temperature increases.

**Methods:**

Thirteen participants (7 relapsing–remitting MS (MS) patients with unilateral (left) optic neuritis and heat-sensitive visual symptoms; 6 controls) underwent visual performance testing on each eye at baseline and during passive heating (0.6℃ rise in gastrointestinal temperature (ΔT_GI_) via a hot water-perfused suit) under two counterbalanced crossover ordered conditions: 1) cold packs (0℃—CLD) or 2) hot packs (50℃—HOT) applied to the lower back. WBTS, visual symptoms, multifocal visual evoked potentials (mf-VEPs) amplitude/latency, and contrast sensitivity were assessed.

**Results:**

ΔT_GI_ was consistent across trials (*p* = 0.213; η_p_^2^ = 0.21). WBTS was only marginally lower (*p* = 0.017; η_p_^2^ = 0.42) in CLD than HOT for MS (CLD: 5.8 ± 0.9 a.u.; HOT: 6.4 ± 0.7 a.u.) and controls (CLD: 5.0 ± 0.9 a.u.; HOT: 5.9 ± 0.7 a.u.). Passive heating worsened (*p* = 0.027; η_p_^2^ = 0.59) visual symptoms in the affected eye similarly (*p* = 0.356; η_p_^2^ = 0.14) for HOT and CLD conditions. Heating reduced mf-VEPs amplitude in the left (affected) eye (*p* = 0.007; η_p_^2^ = 0.50) similarly (*p* = 0.332; η_p_^2^ = 0.09) across groups and conditions. For the unaffected (right) eye, reductions in mf-VEPs amplitude were greater in MS than controls (*p* = 0.031; η_p_^2^ = 0.36), with no difference between conditions (*p* = 0.339; η_p_^2^ = 0.08). mf-VEPs latency and contrast sensitivity were unaffected by heating.

**Conclusion:**

Localised skin cooling during passive heating to a moderate core temperature produces only a modest reduction in WBTS and does not mitigate heat-induced visual performance decrements. The limited perceptual difference achieved suggests the localised skin cooling was insufficient to meaningfully isolate the effects of skin temperature from core temperature.

## Introduction

Up to 80% of people with multiple sclerosis (MS) experience temporary symptom worsening and performance declines in the heat, known as Uhthoff’s phenomenon (Uhthoff 1890). Symptoms like fatigue, blurred vision, and limb weakness emerge or worsen with a core temperature increase of ~ 0.5 °C (Davis 1966; Ollivier 1823; Uhthoff 1890), though some report effects at rises as small as 0.2 °C (Frohman et al. 2013). This heat sensitivity limits participation in exercise, daily activities, and work (Julian et al. 2008). Avoiding heat and physical activity is problematic, as exercise improves strength, balance, fitness, and mental health, delaying MS progression and supporting independence and employment (Julian et al. 2008; Motl et al. 2005, 2009, 2014; Motl & Pilutti 2012, 2016).

Temperature-related changes in axonal conduction are thought to cause the sudden physical decline in people with MS exposed to heat. Increased axonal temperature reduces conduction amplitude, leading to conduction block (Eliasson et al. 1986; Gasser 1931; Tasaki & Fujita 1948). Demyelinated axons develop reversible conduction block at lower temperatures than myelinated axons (F. A. Davis & Jacobson 1971; Rasminsky 1973), with greater demyelination linked to lower blocking temperatures (Schauf & Davis 1974).

Visual symptoms and performance reductions are common in MS (S. L. Davis et al. 2018). Vision is crucial for exteroception, equilibrioception, and proprioception and can significantly impact balance, fall risk, and functional tasks (Lee 1978). Heat exacerbates visual symptoms in MS, with a 0.8 °C rise in gastrointestinal temperature worsening internuclear ophthalmoparesis (INO) (Davis et al. 2008), characterized by slowed eye movements toward the nose (Zee 1992). However, related thermal sensation, symptom severity, and neural conduction measures were not reported (Davis et al. 2008).

Another common condition of the eyes in MS is optic neuritis, an inflammatory demyelinating disorder of the optic nerve, affecting ~ 70% of people with MS, often causing blurred vision, typically in one eye, while also being heat-sensitive (Balcer 2006; Toosy et al. 2014). Studies using unifocal visual evoked potentials (VEPs) to assess heat effects on optic nerve conduction amplitude in MS relative to controls show mixed results (Bajada et al. 1980; Kazis et al. 1982; Matthews et al. 1979; Regan et al. 1977; Saul et al. 1995; Tan 1994), possibly due to the limited sensitivity of unifocal VEPs. To our knowledge, multifocal VEPs (mf-VEPs), which are more sensitive than unifocal VEPs (Fortune & Hood 2003; Grippo et al. 2006; Grover et al. 2008), have not yet been used to evaluate heat sensitivity in MS patients with optic neuritis.

Heat-related reductions in physical performance in MS are often linked to a critical rise in core temperature (Davis et al. 2008; Frohman et al. 2013; Guthrie & Nelson 1995; Ollivier 1823; Uhthoff 1890). However, some studies suggest skin temperature alone can worsen symptoms, like increased postural sway (Poh et al. 2017) and visual or motor issues (Guthrie 1951), even without core temperature changes. Interventions that can alter perception of heat stress, such as cold-water ingestion (Chaseling et al. 2018) and localised skin cooling (Grahn et al. 2008), mitigate symptom worsening and improve performance in people with MS despite similar core temperature rises.

Localised skin cooling of the neck, head and/or wrists is commonly suggested to mitigate heat-related reductions in physical performance by major MS organisations. However, localised skin cooling to the lower back has been shown to elicit colder ratings of thermal sensation relative to all other skin torso regions (Filingeri et al. 2014). Localised skin cooling of the lower back may be more effective at inducing the perception of cooling without a change in core temperature relative to other local skin areas during heat stress. Given the distance from the optic nerve, local skin cooling of the lower back is also unlikely to affect the temperature in that region.

The variability of heat sensitivity in MS has ruled it out as a diagnostic tool (Guthrie & Nelson 1995). Moreover, the effectiveness of some cooling interventions, despite similar core temperatures (Chaseling et al. 2018; Grahn et al. 2008), suggests a psychophysiological role in heat stress perception and symptom worsening. Assessing visual performance during passive heat stress in MS patients may help clarify the impact of skin cooling independent of core temperature changes. This insight could inform more targeted interventions to reduce physical activity avoidance and delay the progression of physical decline in MS.

The aim of this study was to examine whether reductions in WBTS with lower back skin cooling mitigate decrements in visual performance independently of moderate rises in core temperature in people with MS, optic neuritis and heat-sensitive visual symptoms. We hypothesised that reductions in WBTS with lower back skin cooling would mitigate decrements in visual performance independently of moderate rises in core temperature.

## Methods

### Participant eligibility

Participants were excluded from the study if they had a history of respiratory, metabolic or cardiovascular disease, seizures or gastric surgery or were pregnant or had any other ocular or ophthalmological diagnosis at the time of recruitment. MS participants were included in the study if they had unilateral optic neuritis and self-reported heat-sensitive visual symptoms confirmed by a Neurologist but were excluded if they had an exacerbation of visual symptoms unrelated to heat stress within the previous three months or were currently on steroid treatment.

### Participants

Participants voluntarily provided written informed consent prior to commencing the study. The study received prior approval from the University of Sydney Human Research Ethics Committee (File #2018/791) and conformed to the principles set forth in the Declaration of Helsinki 2013, except for registration in a clinical trials database. Seven participants (5 women, 2 men) with Relapsing–Remitting Multiple Sclerosis (RRMS), an Expanded Disability Severity Scale (EDSS) ≤ 3.5, optic neuritis in the left eye and self-reported heat-sensitivity affecting vision in the left eye (confirmed by a Neurologist) with a mean age of 35 ± 9 year, body mass of 70 ± 11 kg and height of 170 ± 6 cm and 6 healthy control participants (4 women, 2 men) with a mean age of 29 ± 9 year, body mass of 65 ± 12 kg and height of 174 ± 11 cm completed the study. One control and three MS participants wore prescription glasses, and one MS participant wore prescription contact lenses.

### Experimental design

**Fig. 1:**
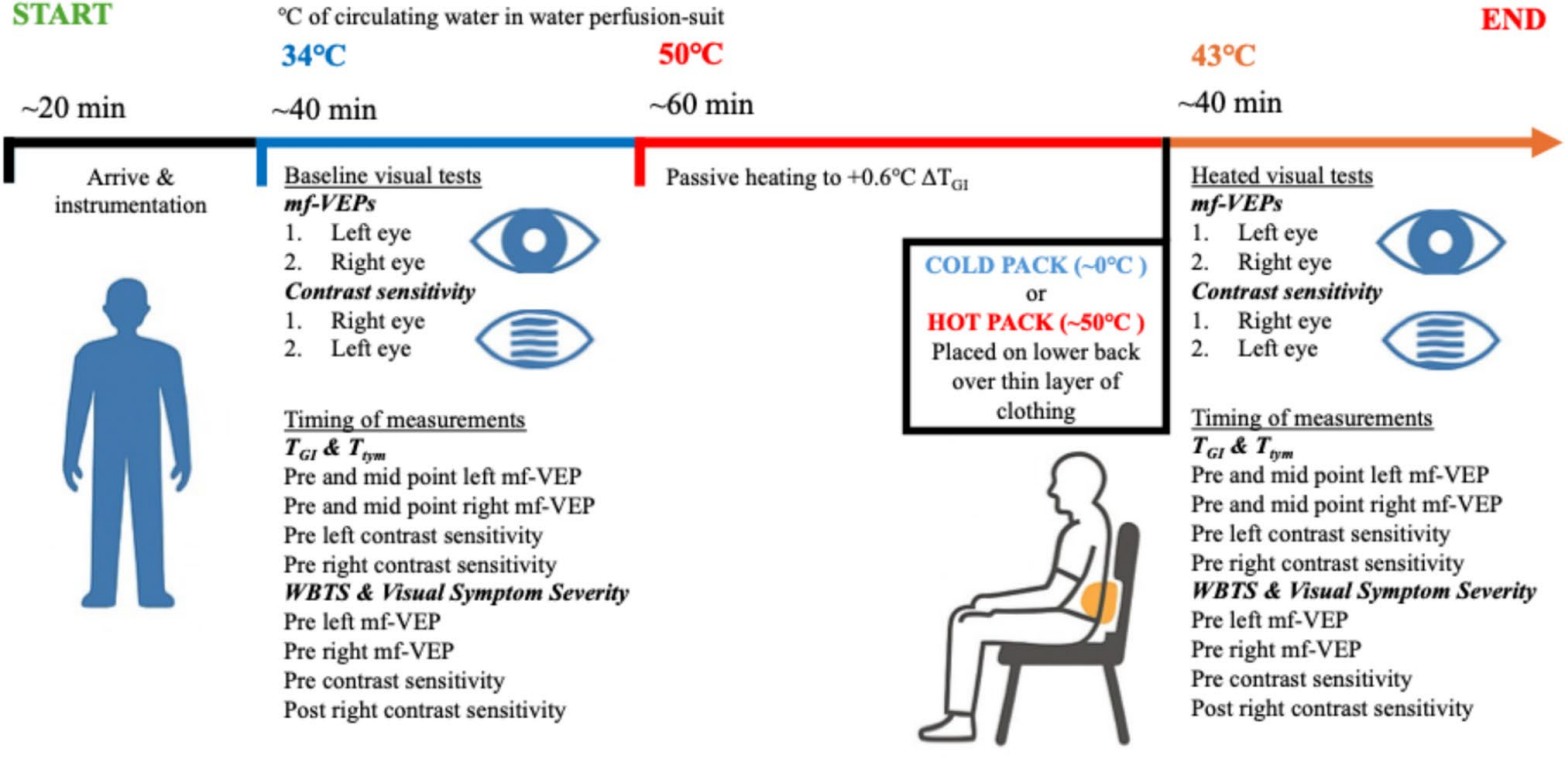
Experimental protocol timeline. mf-VEPs: multifocal visual evoked potentials. T_GI_, gastrointestinal temperature. T_tym_, tympanic temperature. WBTS, whole body thermal sensation

Participants visited the Brain and Mind Centre at the University of Sydney, Australia, on a total of 2 separate occasions during which the experimental protocol (Fig. 1) was completed with two thermal packs (Reusable Hot and Cold Pack, 250 cm^2^, Livingstone, Mascot, NSW, Australia) at a surface temperature of 1) ~ 50℃ (HOT); or 2) ~ 0℃ (CLD) placed on the lower back for the visual tests in the heated stage of the experimental protocol. Participants were blinded to the intervention (prior to placement of the hot or cold packs on the back) and study hypothesis. All experimental trials commenced in the morning between 8:00 and 10:00 and were presented using a counterbalanced crossover order design each separated by at least 24 h. Prior to all experimental trials, participants were instructed to abstain from alcohol, caffeine and strenuous exercise for 24 h. Participants wore leggings or exercise shorts and a t-shirt or singlet with a bandage wrapped around the torso and over the t-shirt/singlet and were instructed to wear the same ensemble for each trial. The bandage allowed quick application of the thermal packs once the change in gastrointestinal temperature (ΔT_GI_) was ~ 0.6℃, whilst the clothing provided a thin barrier to prevent cold burns. Once instrumented, participants donned the water perfusion-suit (One-piece Coretec, Delta Temax, Inc., Pembroke, ON, Canada).

### Experimental protocol

Participants were in an upright seated position throughout the duration of the experiment which was completed in a temperature regulated room (~ 21 ℃). Participants completed visual tests (Fig. 1) at baseline gastrointestinal temperature (T_GI_) and heated to approximately + 0.6℃ ΔT_GI_. An increase in core temperature of 0.8℃ (range: 0.6 ℃ to 1.1 ℃) was used previously to model Uhthoff’s phenomenon (Davis et al. 2008), however a large body of evidence indicates a rise in core temperature of ≥ 0.5 ℃ is sufficient to induce clinically significant symptom worsening (Davis 1966; Frohman et al. 2013; Guthrie & Nelson 1995; Ollivier 1823; Uhthoff 1890). Steady-state core and skin temperature were maintained while the visual tests were completed. Maintaining baseline body temperatures was achieved by circulating 34℃ water through the water perfusion-suit. To heat the participant, 50 ℃ water was perfused through the suit with the participant cloaked in a reflective blanket. When + 0.6 ℃ ΔT_GI_ was achieved the temperature of the water perfusing the suit was changed to 43 ℃ to maintain raised T_GI_ (determined with pilot testing). Thermal packs were then placed on the lower back between the bandage and clothing with the back of the suit rolled up and pinned together to prevent it falling over the thermal packs. While cooling the head and neck region is effective at lowering thermal sensation (Nakamura et al. 2013), it was not viable when assessing visual performance since the face and eyes need to be clear of distraction to ensure clear visual focus. Cooling the head and/or neck could also interfere with the mf-VEP electrodes. T_GI_ and tympanic temperature (T_tym_) were recorded 6 occasions throughout visual testing (4 points: pre- and mid-point left and pre- and mid-point right mf-VEP tests; 2 points: pre left and pre right contrast sensitivity tests) and perceptual measures were recorded 4 occasions throughout visual testing (2 points: pre left and pre right mf-VEPs; 2 points: pre and post contrast sensitivity tests) (Fig. 1).

### Visual performance protocol

Visual tests in order of testing included: 1. Left, then right eye mf-VEPs and 2. Right, then left eye contrast sensitivity (Fig. 1). Mf-VEPs testing was completed in dimmed room lighting and contrast sensitivity was completed with the room lights off. The contralateral eye to the tested eye was covered through each test.

### Instrumentation

*Body temperatures:* T_GI_ was measured using a CorTemp Ingestible Core Body Temperature Sensor (HQ, Inc., Palmetto, FL, USA) that was swallowed by the participant before bed the night before an experimental trial. T_tym_ was measured using a Mon-a-therm general-purpose thermistor probe 400TM (Covidien, Mansfield, MA, USA) self-inserted into the aural canal until resting near the tympanic membrane and then insulated with cotton wool and ear defenders. Skin temperature was measured using wireless Thermochron iButtons (DS1922-F5#, Embedded Data System, Lawrenceberg, KY, USA) secured to the chest, shoulder, thigh, calf and lower back using hypoallergenic surgical tape (3 M Transpore 1527–1). Mean skin temperature (T_sk_) was estimated using a 4-point weighted mean according to Ramanathan (Ramanathan 1964) and lower back skin temperature (T_LBsk_) was reported separately.

*Perceptual measures:* WBTS was assessed using a bipolar visual analogue scale with 7 equally spaced anchor points along a 100 mm scale; “cold”, “cool”, “slightly cool”, “neutral”, “slightly warm”, “warm” and “hot”. Participants were instructed to “*select one of the seven anchor points that best describes their current WBTS.*” Left eye (optic neuritis) visual symptom severity was rated using the same 100 mm scale with 4 equally spaced anchor points; “none”, “mild”, “moderate” and “severe”. This scale was used to report any change in vision in the left eye of MS participants, including, but not limited to blurring, fogging, colour blindness, phosphenes (ring or spot of light in vision) or the Pulfrich effect (images in motion creating depth or a three-dimensional effect) (Toosy et al. 2014). Participants were instructed to “*select one of the four anchor points that best describes their current visual symptom severity*.”

**Fig. 2:**
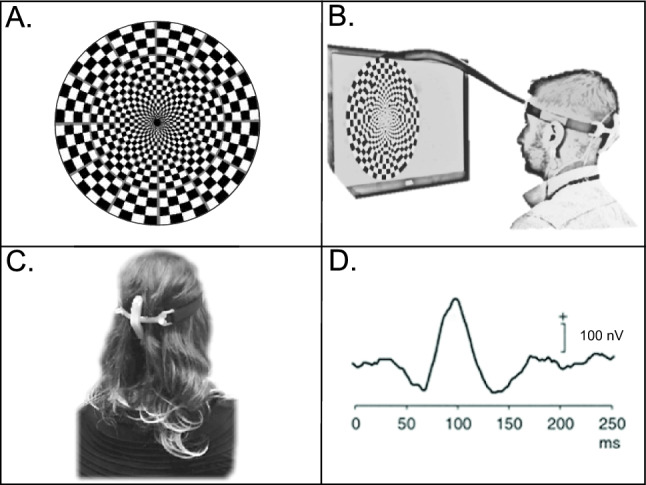
Multifocal Visual Evoked Potentials (mf-VEPs) testing. **A.** Alternating checkerboard pattern-reversal stimuli within a circle. **B.** Pattern reversal stimuli presented on a screen 30 cm from participant. **C.** Electrode set-up on the occiput. **D.** Example of a visual evoked potential

*Mf-VEPs* measures neural conductance through the optic nerve to the visual cortex. Participants are presented with alternating pattern-reversal stimuli composed of checkerboard patterns within a circle (Fig. 2A) presented on a screen 30 cm from the participant (Fig. 2B). The checkerboard pattern reverses in a pseudorandom manner dependent on a binary m-sequence. The participant focuses on a target at the centre of the circle and the alternating checkboard patterns stimulate mf-VEPs (Fig. 2D) in the visual cortex detected by carefully placed electrodes on the occiput (back of the head) (Fig. 2C).

The VisionSearch1 system (Vision Search Pty. Ltd., Sydney, NSW, Australia) was used for mf-VEPs testing. Participant’s hair was parted, and the scalp abraded with *nuprep* abrasive gel (Weaver and Company, Aurora, CO, USA) for each of the four gold cup electrodes. Electrodes were placed on the participant’s occiput in the form of a cross using a custom-built silicon electrode holder with a grounding electrode-clip on the ear lobe. This set-up allows recording of neural signal at the visual cortex in two orthogonal channels (vertical and horizontal). When each channel displayed low electrical resistance (< 10 Ω) between electrodes, medical grade adhesive (Ten 20, Weaver and Company, Aurora, CO, USA) was placed around electrodes to hold them in place. High conductance SignaGel electrode gel (Parker Laboratories, Inc., Fairfield, NJ, USA) was then squeezed into each electrode. Test runs took 110 s with ~ 30 s rest between each run and repeated until there was < 15% variance in signal compared to the average of previous test runs. The range of test runs needed was 4 to 10 for each eye. The “progression analysis” tool in a custom build of the VisionSearch1 software was used to report absolute average mf-VEPs amplitude (signal strength) in nanovolts (nV) and latency (length of time of signal) in milliseconds (ms) for all sectors as previously described (Klistorner et al. 2018).

**Fig. 3:**
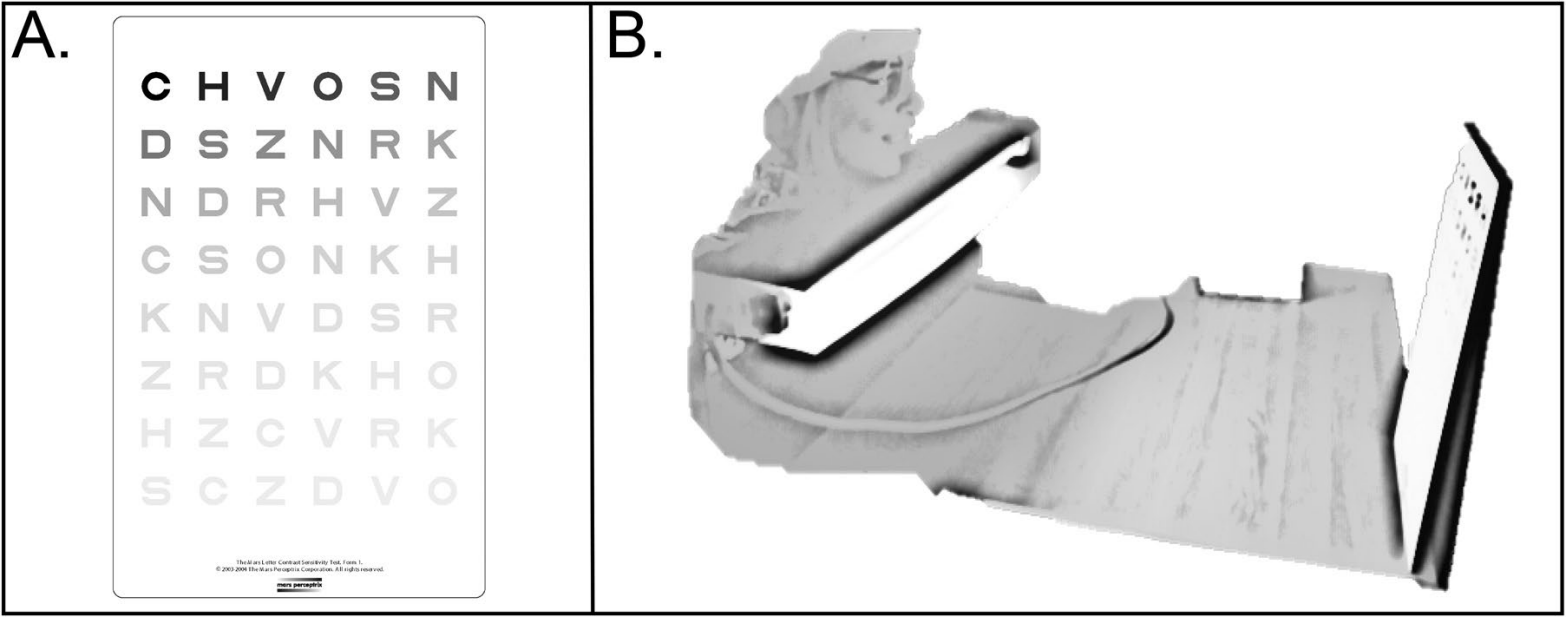
Contrast sensitivity testing. **A.** Form 1 of the Mars Letter Contrast Sensitivity Test. **B.** Experimental set-up

*Contrast sensitivity* was assessed to determine the capacity for the visual system to differentiate objects from the background and was performed using the Mars Letter Contrast Sensitivity Test (The Mars Perceptrix Corporation, Chappaqua, NY, USA). The test comprises a set of three charts for testing peak visual contrast sensitivity with letters progressively fading in contrast (Fig. 3A). A higher contrast sensitivity demonstrates better vision with the observer better seeing the difference between light and dark contrast. Each chart was presented separately and uniformly illuminated with a lamp with the participant resting their chin on the lamp approximately 50 cm from the chart (Fig. 3B). Participants were instructed to read the letters from left to right across each line of the chart and the test was terminated when the patient made two consecutive errors or reached the end of the chart. The log contrast sensitivity score was given for each chart following The Mars Letter Contrast Sensitivity Test User Manual (19th Feb 2013) and the average log contrast sensitivity score for the 3 charts was analysed.

### Statistical analysis

Body temperatures and perceptual measures were averaged for each experimental stage (i.e., baseline visual testing, heated visual testing) and change in body temperature was calculated from the final measure taken at baseline.

Dependent variables of mf-VEPs amplitude, mf-VEPs latency and contrast sensitivity for the left and right eye and T_GI_, ΔT_GI_, T_tym_, ΔT_tym_ T_sk_, ΔT_sk_, T_LBsk_, ΔT_LBsk_ and WBTS were assessed using a three-way analysis of variance (ANOVA) employing the independent variables of group (2 levels: MS and Control), experimental stage (2 levels: Baseline and Heated) and condition (2 levels: HOT and CLD). The dependent variable of left eye (optic neuritis) visual symptom severity was assessed using a two-way ANOVA employing the independent variables of condition (2 levels: HOT and CLD) and experimental stage (2 levels: Baseline and Heated). A two-way ANOVA was used to assess mf-VEPs amplitude, mf-VEPs latency and contrast sensitivity in MS participants employing the independent variables of eye (2 levels: Left and Right) and experimental stage (2 levels: Baseline and Heated) within each experimental trial.

All analyses included a Mauchly's test for sphericity and applied a Greenhouse–Geisser correction factor if required. If a significant interaction was observed, post-hoc comparisons were conducted using a Bonferroni multiple comparisons test. Where values were missing, data were analysed by fitting a mixed model. A critical alpha level error of 0.05 was maintained throughout. All data are presented as mean ± standard deviation (SD). Statistical analyses were conducted using GraphPad Prism Version 8.3.1 for Windows (Graphpad Software, La Jolla, CA, USA).

## Results

### Body temperatures

In 2 cases, an MS participant excreted the telemetric temperature pill prior to the second experimental trial, so ΔT_tym_ and timing from the first trial was used to match heat stress exposure. Hence, *n* = 6 for the MS group for T_GI_ and ΔT_GI_. By design, T_GI_ (η_p_^2^ = 0.97), ΔT_GI_ (η_p_^2^ = 0.99)_,_ T_tym_ (η_p_^2^ = 0.95), ΔT_tym_ (η_p_^2^ = 0.94), T_sk_ (η_p_^2^ = 0.98) and ΔT_sk_ (η_p_^2^ = 0.98) were all higher (*p* < 0.001) when passively heated and were not altered by the application of hot and cold packs, nor different between MS and CON (T_GI_: *p* = 0.329, η_p_^2^ = 0.14; ΔT_GI_: *p* = 0.213, η_p_^2^ = 0.21; T_tym_: *p* = 0.372, η_p_^2^ = 0.07; ΔT_tym_: *p* = 0.526, η_p_^2^ = 0.04; T_sk_: *p* = 0.816, η_p_^2^ = 0.01_;_ ΔT_sk_: *p* = 0.814, η_p_^2^ = 0.01) (Table 1).

**Table 1:** Body temperatures at baseline and heated experimental stages for MS (n = 7) and control (n = 6) participants with hot packs (HOT) or cold packs (CLD) applied to the lower back

Body	HOT				CLD			
temperature	MS		Control		MS		Control	
(℃)	Baseline	Heated	Baseline	Heated	Baseline	Heated	Baseline	Heated
T_GI_	37.2 ± 0.4^a^	37.7 ± 0.5^a,^^c^	37.1 ± 0.3	37.7 ± 0.3^c^	37.1 ± 0.5^a^	37.6 ± 0.4^a,^^c^	37.2 ± 0.3	37.7 ± 0.3^c^
ΔT_GI_	–	0.6 ± 0.1^a,^^c^	–	0.6 ± 0.1^c^	–	0.6 ± 0.1^ca^	–	0.6 ± 0.1^c^
T_tym_	36.0 ± 0.4	37.0 ± 0.4^c^	35.9 ± 0.3	36.9 ± 0.3^c^	35.7 ± 0.7	37.0 ± 0.4^c^	35.8 ± 0.3	36.9 ± 0.4^c^
ΔT_tym_	–	1.3 ± 0.4^c^	–	0.9 ± 0.2^c^	–	1.0 ± 0.3^c^	–	0.9 ± 0.3^c^
T_sk_	33.7 ± 0.5	37.2 ± 0.4^c^	33.9 ± 0.4	37.1 ± 0.6^c^	33.9 ± 0.4	37.3 ± 0.4^c^	33.9 ± 0.4	37.0 ± 0.4^c^
ΔT_sk_	–	3.2 ± 0.7^c^	–	3.0 ± 0.5^c^	–	3.1 ± 0.7^c^	–	2.9 ± 0.5^c^
T_LBsk_	34.6 ± 1.0	37.8 ± 1.9^b^	35.1 ± 0.4	38.2 ± 1.0 ^b^	35.1 ± 0.4	22.7 ± 8.6^b^	35.4 ± 0.3	17.5 ± 4.4^b^
ΔT_LBsk_	–	3.2 ± 1.2^b^	–	3.0 ± 1.2^b^	–	−12.5 ± 8.7^b^	–	−18.0 ± 4.5^b^

T_GI_, gastrointestinal temperature; ΔT_GI_, change in gastrointestinal temperature; T_tym_, tympanic temperature; ΔT_tym_, change in tympanic temperature; T_sk_, mean skin temperature; ΔT_sk_, change in mean skin temperature; T_LBsk_, lower back skin temperature; ΔT_LBsk_, change in lower back skin temperature

^a^*n* = 6

^b^*p* < 0.001 Experimental stage-by-condition interaction

^c^*p* < 0.001 Main effect of heating. Values are mean ± SD. All measures were analysed using a three-way ANOVA employing the independent variables of group (2 levels: MS and Control), experimental stage (2 levels: Baseline and Heated) and condition (2 levels: HOT and CLD)

By design, T_LBsk_ was lower (*p* < 0.001; η_p_^2^ = 0.82) with cold packs relative to hot packs, with no difference observed between groups (*p* = 0.178; η_p_^2^ = 0.16) (Table 1). ΔT_LBsk_ was lower from baseline with cold packs applied compared to the higher ΔT_LBsk_ with hot packs applied (*p* < 0.001; η_p_^2^ = 0.90) in both groups (*p* = 0.171; η_p_^2^ = 0.16) (Table 1).

### Perceptual measures

**Fig. 4:**
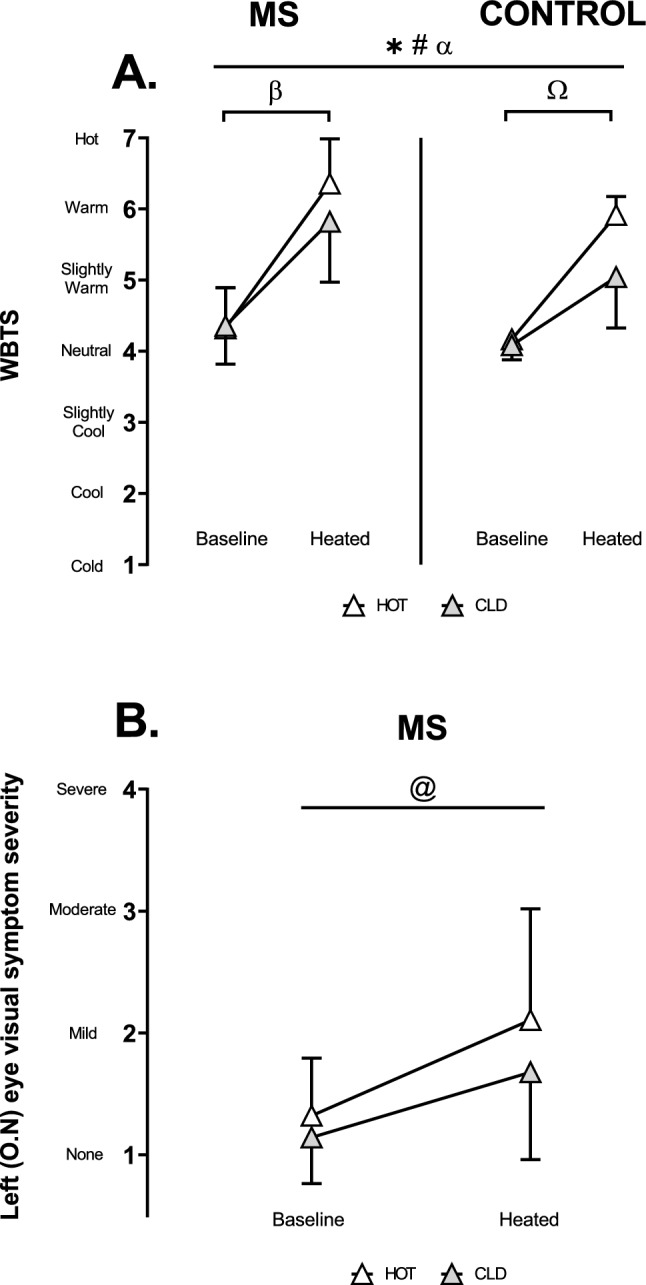
Average perceptual measures for baseline and heated experimental stages. **A.** Whole-body thermal sensation (WBTS) in MS (*n* = 7) and control (*n* = 6) participants. **B.** Left eye visual symptom severity in MS (*n* = 7) participants. O.N: Optic neuritis. α (*p* = 0.017) Experimental stage-by-condition interaction. *(*p* < 0.001) Main effect of heating. #(*p* = 0.001) Main effect of CLD. β (*p* = 0.014) Ω (*p* < 0.001) Baseline vs heated in HOT. @ (*p* = 0.027) Main effect of heating. Values are mean ± SD. WBTS was analysed using a three-way ANOVA employing the independent variables of group (2 levels: MS and Control), experimental stage (2 levels: Baseline and Heated) and condition (2 levels: HOT and CLD). Left eye visual symptom severity in MS participants was analysed using a two-way ANOVA employing the independent variables of condition (2 levels: HOT and CLD) and experimental stage (2 levels: Baseline and Heated)

WBTS was only marginally lower (*p* = 0.017; η_p_^2^ = 0.42) with cold packs relative to hot packs in both groups when passively heated (Fig. 4A). Multiple comparisons showed heat increased WBTS in both groups in the HOT condition (MS: *p* = 0.014, *d* = 3.62; controls: *p* < 0.001, *d* = 11.07) (Fig. 4A). In the MS group, passive heating worsened visual symptoms in the left (optic neuritis) eye (*p* = 0.027; η_p_^2^ = 0.59) similarly between HOT and CLD conditions (*p* = 0.356; η_p_^2^ = 0.14) (Fig. 4B).

### Visual performance

#### Mf-VEPs amplitude

**Fig. 5:**
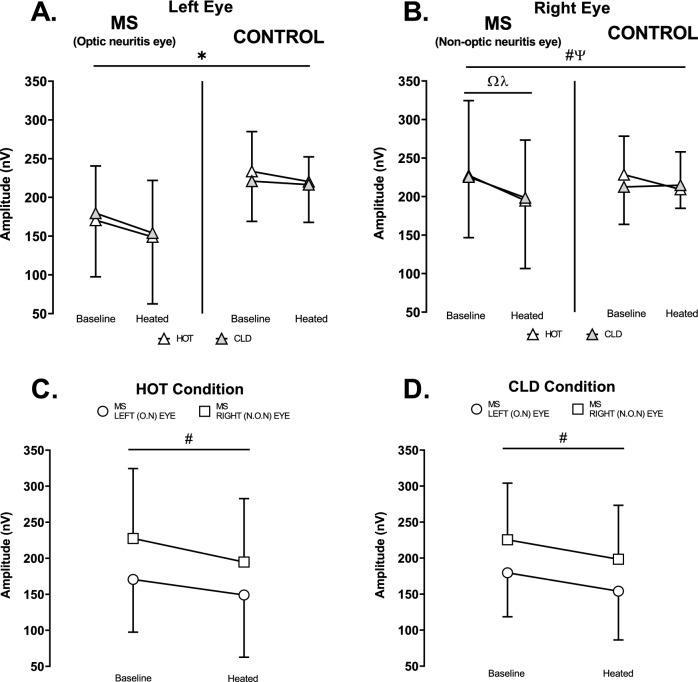
Multifocal Visual Evoked Potentials Amplitude (nV) at baseline and heated. **A.** Left eye HOT vs CLD in MS (O.N eye) (*n* = 7) and control (*n* = 6). **B.** Right eye HOT vs CLD in MS (N.O.N eye) (*n* = 7) and control (*n* = 6). **C.** HOT condition left eye (O.N) vs right eye (N.O.N) in MS (*n* = 7). **D.** CLD condition left eye (O.N) vs right eye (N.O.N) in MS (*n* = 7). O.N: Optic neuritis. N.O.N: Non-optic neuritis. Ψ (*p* = 0.031) Experimental stage-by-group interaction. * (*p* = 0.007) # (*p* = 0.001) Main effect of heating. Ω (*p* = 0.003) Baseline VS Heated in HOT. λ (*p* = 0.021) Baseline VS Heated in CLD. Values are mean ± SD. Mf-VEP amplitude in graphs A and B were analysed using a three-way ANOVA employing the independent variables of group (2 levels: MS and Control), experimental stage (2 levels: Baseline and Heated) and condition (2 levels: HOT and CLD). Mf-VEP amplitude in graphs C and D were analysed using a two-way ANOVA employing the independent variables of eye (2 levels: Left and Right) and experimental stage (2 levels: Baseline and Heated)

Passive heating reduced mf-VEPs amplitude in the left eye (*p* = 0.007; η_p_^2^ = 0.50) similarly for both groups (*p* = 0.163; η_p_^2^ = 0.17) with no difference between HOT and CLD conditions (*p* = 0.332; η_p_^2^ = 0.09) (Fig. 5A). In the right eye, passive heating reduced mf-VEPs amplitude in MS relative to controls (*p* = 0.031; η_p_^2^ = 0.36) with no difference between cold and hot packs (*p* = 0.339; η_p_^2^ = 0.08). Multiple comparisons showed heat reduced the amplitude in the right eye of MS participants in the HOT (*p* = 0.003; d = 3.32) and CLD (*p* = 0.021; d = 2.28) conditions but not in controls (HOT: *p* = 0.593, d = 0.99; CLD: *p* > 0.999, d = 0.13) (Fig. 5B).

The heat reduced amplitude in both eyes of MS participants in the HOT (*p* = 0.001; η_p_^2^ = 0.89) (Fig. 5C) and CLD (*p* = 0.001; η_p_^2^ = 0.88) (Fig. 5D) conditions with minimal difference between left and right eyes (HOT: *p* = 0.207, η_p_^2^ = 0.25; CLD: *p* = 0.868, η_p_^2^ = 0.01) (Fig. 5C and D).

#### Mf-VEPs latency

**Fig. 6:**
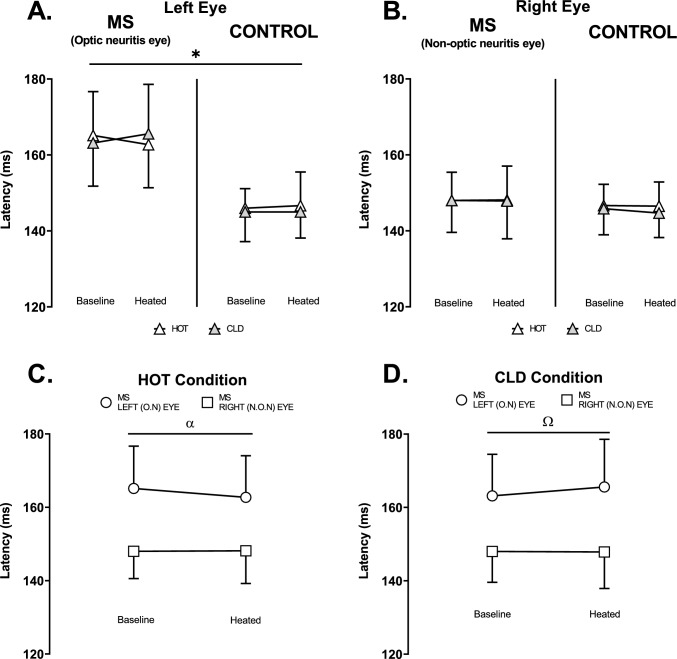
Multifocal visual evoked potentials latency (ms) at baseline and heated (ΔT_GI_: 0.6 ± 0.1℃). **A.** Left eye HOT vs CLD in MS (O.N eye) (*n* = 7) and control (*n* = 6). **B.** Right eye HOT vs CLD in MS (N.O.N eye) (*n* = 7) and control (*n* = 6). **C.** HOT condition left eye (O.N) vs right eye (N.O.N) in MS (*n* = 7). **D.** CLD condition left eye (O.N) vs right eye (N.O.N) in MS (*n* = 7). O.N: Optic neuritis. N.O.N: Non-optic neuritis. *(*p* = 0.006) Main effect of MS (O.N). α (*p* = 0.001) Ω (*p* = 0.002) Main effect of left eye VS right eye. Values are mean ± SD. Mf-VEP latency in graphs A and B were analysed using a three-way ANOVA employing the independent variables of group (2 levels: MS and Control), experimental stage (2 levels: Baseline and Heated) and condition (2 levels: HOT and CLD). Mf-VEP latency in graphs C and D were analysed using a two-way ANOVA employing the independent variables of eye (2 levels: Left and Right) and experimental stage (2 levels: Baseline and Heated)

In the left eye, MS had a greater mf-VEPs latency relative to controls (*p* = 0.006; η_p_^2^ = 0.51) which was unaltered by passive heating and the application of hot and cold packs (*p* = 0.073; η_p_^2^ = 0.26) (Fig. 6A). In the right eye, latency did not reveal any main effects or interactions (Fig. 6B).

There was greater latency in the left eye compared to the right eye in MS participants in the HOT (*p* = 0.001; η_p_^2^ = 0.88) (Fig. 6C) and CLD (*p* = 0.002; η_p_^2^ = 0.83) (Fig. 6D) condition with minimal effect of heating in each condition (HOT: *p* = 0.074, η_p_^2^ = 0.44; CLD: *p* = 0.111, η_p_^2^ = 0.37) (Fig. 6C and D).

#### Contrast sensitivity

**Fig. 7:**
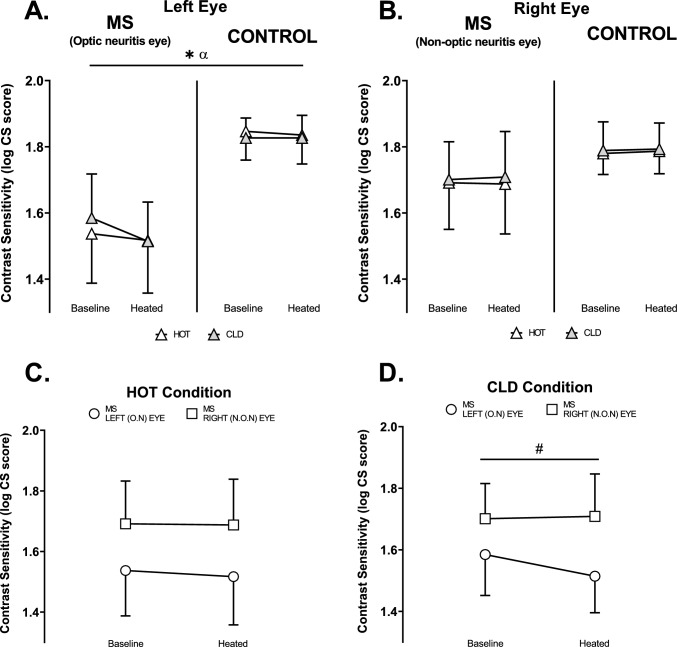
Contrast sensitivity (log CS score) at baseline and heated **A.** Left eye in MS (*n* = 7) and control (*n* = 6). **B.** Right eye in MS (*n* = 7) and control (*n* = 6). **C.** HOT condition left eye (O.N) vs right eye (N.O.N) in MS (*n* = 7). **D.** CLD condition left eye (O.N) vs right eye (N.O.N) in MS (*n* = 7). O.N: Optic neuritis. N.O.N: Non-optic neuritis. α (*p* = 0.049) Experimental stage-by-group-by-condition interaction. *(*p* < 0.001) Main effect of MS (O.N). #(*p* = 0.038) Main effect of left eye vs right eye. Values are mean ± SD. Contrast sensitivity in graphs A and B were analysed using a three-way ANOVA employing the independent variables of group (2 levels: MS and Control), experimental stage (2 levels: Baseline and Heated) and condition (2 levels: HOT and CLD). Contrast sensitivity in graphs C and D were analysed using a two-way ANOVA employing the independent variables of eye (2 levels: Left and Right) and experimental stage (2 levels: Baseline and Heated)

MS had a lower contrast sensitivity in the left eye relative to controls (*p* < 0.001; η_p_^2^ = 0.70) (Fig. 7A). The application of cold packs when passively heated reduced contrast sensitivity in MS relative to controls in the left eye (*p* = 0.049; η_p_^2^ = 0.31) (Fig. 7A). In the right eye, latency did not reveal any main effects or interactions (Fig. 7B).

Contrast sensitivity was lower in the left eye compared to the right eye in MS participants in the CLD (*p* = 0.038; η_p_^2^ = 0.54) (Fig. 7D) but not the HOT (*p* = 0.068; η_p_^2^ = 0.45) (Fig. 7C) condition with no effect of heating in either condition (HOT: *p* = 0.660, η_p_^2^ = 0.03; CLD: *p* = 0.114, η_p_^2^ = 0.36) (Fig. 7C and D).

## Discussion

This study aimed to determine whether lowering WBTS with the application of cold packs on the lower back during passive heat stress mitigated decrements in visual performance in people with MS, optic neuritis and heat-sensitive visual symptoms independently of moderate rises in core temperature of ~ 0.6 ℃. While WBTS was statistically lower in the cold condition (Fig. 4A), the perceptual difference was only modest, and participants continued to report feeling “slightly warm” or “warm” in both trials. This small change in thermal sensation did not mitigate the heat-related worsening of visual symptoms (Fig. 4B) nor the decline in mf-VEP amplitude (Fig. 5). To our knowledge, this is the first study to attempt isolating the perceptual effects of localised skin cooling at a fixed, raised core temperature while assessing visual performance and heat-sensitive visual symptoms in this population. However, the limited reduction in WBTS constrained our ability to draw firm conclusions regarding the independent role of skin temperature, and hence WBTS in Uhthoff’s phenomenon.

### Perceptions of whole-body thermal sensation and visual symptoms

Skin cooling of the lower back with ~ 0 ℃ ice packs only slightly reduced WBTS in MS (CLD: 5.8 ± 0.9 a.u. vs HOT: 6.4 ± 0.7 a.u.) and controls (CLD: 5.0 ± 0.9 a.u. vs HOT: 5.9 ± 0.7 a.u.) without altering the rise in core temperature (ΔT_GI_: ~ 0.6 ℃). This weak intervention was unsurprisingly also ineffective at reducing heat-related visual symptom worsening in MS. As such, we were unable to meaningfully manipulate WBTS and therefore could not adequately test our hypothesis that reducing WBTS through localised skin cooling would mitigate visual performance decrements independently of core temperature. Nonetheless, we believe this hypothesis remains plausible and supported by previous (Beenakker et al. 2001; Feys et al. 2005; Grahn et al. 2008; Meyer-Heim et al. 2007; Reynolds et al. 2011) and emerging literature (Buoite Stella et al. 2020; Christogianni et al. 2023) suggesting that changes in skin temperature and thermal perception—without accompanying changes in core temperature—can influence heat-sensitive symptom expression in people with MS. For instance, Christogianni et al. demonstrated that perceptual worsening of fatigue occurred during passive heat stress in a climatic chamber, with manipulations of mean skin temperature alone (Christogianni et al. 2023). Similarly, improvements in walking distance have been observed when participants reported “neutral” thermal sensations while wearing a torso cooling garment, compared to warmer sensations from a sham vest, although core temperature was not measured (Buoite Stella et al. 2020).

The selection of a homogeneous MS population (with both unilateral optic neuritis and heat-sensitive visual symptoms) and the use of passive heating allowed us to isolate the visual pathway in the central nervous system (CNS) in an attempt to mechanistically test the role of changes in skin temperature and associated perceptions of cooling in heat-related symptom worsening in a controlled manner. Based on previous findings, it may be possible that lowering WBTS with localised skin cooling but no reduction in core temperature provides some transient relief from general fatigue and localised muscle weakness or tremor (Beenakker et al. 2001; Feys et al. 2005; Grahn et al. 2008; Meyer-Heim et al. 2007), but our findings do not offer insight into whether WBTS changes alone can influence demyelinated CNS pathways. Instead, our limited results show the importance of managing increases in core temperature as a key strategy for mitigating heat-related symptom exacerbation in MS, particularly when symptoms are mediated by central neural pathways.

### Visual performance

Mf-VEPs amplitude and latency provided a signal of optic neural conduction that allowed us to investigate the electrophysiological effect of elevations in core temperature and localised skin cooling on the function of the visual system. Moderate rises in core temperature reduced mf-VEPs amplitude in both eyes of MS and control participants (Fig. 5) reflective of the heat-related reduction in conduction amplitude found in in vitro (Eliasson et al. 1986; Gasser 1931; Tasaki & Fujita 1948) and animal studies (F. A. Davis & Jacobson 1971; Rasminsky 1973) and unifocal VEPs studies in MS (Kazis et al. 1982; Matthews et al. 1979; Saul et al. 1995).

A slightly lower WBTS with localised skin cooling had no effect on this heat-related reduction. It is possible that the moderate rise in core temperature caused a heat-related reduction in amplitude in the optic neuritis eye (Fig. 5A) that may have fallen below the theorised axonal safety factor (i.e., the amplitude at which there is enough effective transmission for signalling) (Rutkove 2001). This fall below the safety factor may have blocked conduction in the optic nerve, causing the visual symptoms (Fig. 4B). This finding is supported by the association between greater demyelination and lower blocking temperatures (Schauf & Davis 1974).

There was a heat-related reduction in amplitude of the right non-optic neuritis eye in MS participants relative to controls (Fig. 5B). These data are similar to those found in previous studies whereby a slowly progressive, subclinical (non-symptomatic) optic neuropathy can be evident through lower VEPs amplitude in optic pathways not diagnosed with optic neuritis (Feinsod & Hoyt 1975; Kahana et al. 1973). Our finding may indicate sub-clinical right optic nerve involvement in the MS participants (Feinsod & Hoyt 1975; Kahana et al. 1973), with the conduction amplitude falling, but remaining above the safety factor (Rutkove 2001).

As shown previously (Fraser et al. 2006; Grover et al. 2008), mf-VEP latency was greater (indicating slowing of conduction velocity) in the left optic neuritis eye of MS participants relative to their right non-optic neuritis eye and left eye of controls (Fig. 6) due to demyelination of the optic nerve (Fraser et al. 2006; Grover et al. 2008). It is commonly believed that the heat-related reduction in amplitude and increase in conduction block in demyelinated axons may be related to increased conduction velocity limiting opening time of sodium channels (Hodgkin & Huxley 1945; Hodgkin & Katz 1949; Rutkove 2001). However, latency was unchanged with passive heating in the present study despite the heat-related reduction in amplitude in MS participants (Fig. 5). A heat-related reduction in unifocal VEPs amplitude with no change in latency in MS was reported previously (Matthews et al. 1979). This finding might be related to the proportion and severity of demyelinated axons and axonal loss in the optic nerve (Barton et al. 2019; Toosy et al. 2014). Within a nerve, heat may block transmission in severely demyelinated axons while other more myelinated axons may transmit at a greater velocity without falling below the safety factor (Hodgkin & Huxley 1945; Hodgkin & Katz 1949; Rutkove 2001; Schauf & Davis 1974). The reduction in signal at the visual cortex due to heat-related axonal conduction block in combination with baseline axonal loss may have reduced sensitivity to detect any heat-related change in latency.

Contrast sensitivity was unchanged with passive heating in both MS and controls (Fig. 7), likely due to its lower methodological sensitivity relative to mf-VEPs (Barton et al. 2019). Hence, the effect of a marginally lower WBTS on mitigating heat-related reductions in contrast sensitivity was untested. The cold packs seemingly reduced contrast sensitivity in the optic neuritis eye of MS participants relative to no change with hot packs and no change in controls (Fig. 5A). This observation may be due to a type 1 error. Nevertheless, contrast sensitivity measures were sensitive enough to detect the large effect size of optic neuritis, demonstrating worse contrast sensitivity in the left optic neuritis eye of MS participants compared to controls (Fig. 7A) and their right non-optic neuritis eye (Fig. 7D), which has been shown previously (Balcer 2006; Toosy et al. 2014).

### Limitations

Our findings may be limited by the modest, albeit statistically significant, reductions in WBTS thermal sensation during our CLD trial and moderate rises in core temperature (+ 0.6 ℃) relative to other studies (e.g. + 0.8 ℃ (Davis et al. 2008)). The specific local cooling site was selected due to its high cold sensitivity (Filingeri et al. 2014); however, the absolute skin area was relatively small (0.025 m^2^). Altered sensitivity to local cold stimuli in people with MS (Christogianni et al. 2024) may contribute to individual variability in how local cooling interventions are perceived and whether they result in meaningful changes in WBTS. A greater reduction in WBTS may have been reported by participants if we cooled a larger area of skin such as with a cooling garment covering the torso (Buoite Stella et al. 2020). However, cooling a larger area would have increased conductive heat loss and likely lowered core temperature and potentially undermined our ability to investigate the study hypothesis. Alternatively, the addition of cooling the neck could increase the cold stimulus and although it was not practical in this study, it could be used to assess other outcome measures without violating external validity. Increasing cold sensation with menthol application to a large area of skin may also be a method to elicit a colder WBTS without altering core temperature (Gillis et al. 2010; Peier et al. 2002).

Data collection for this trial ceased with recruitment ending at seven MS participants and six control participants, short of the target of ten participants in each group. We observed an effect size of 1.1 for the reduction in mf-VEPs amplitude in the left eye following heating in the MS participants (−17 ± 15%) compared to control participants (−2 ± 12%). However, this comparison yielded a *p*-value of 0.163, indicating statistical non-significance at the current sample size, with a power of only 44%. Had recruitment been completed, the trial may have achieved > 75% power to detect a significant reduction in mf-VEPs amplitude in the left eye of MS participants relative to controls at the observed effect size. These findings highlight the need for further studies with adequate participant numbers and an intervention that results in a more meaningful reduction in WBTS to confirm the observed trends.

A larger sample size may also reveal a heat-related reduction in contrast sensitivity in the optic neuritis eye of MS participants, reflecting the evident heat-related mf-VEPs amplitude reduction and visual symptom worsening. Localised skin cooling only partially lowered WBTS with minimal (if any) effect on heat sensitivity. However, considering previously reported effects of skin temperature (Buoite Stella et al. 2020; Christogianni et al. 2018, 2023; Guthrie 1951; Poh et al. 2017), further research is required to better identify if there is an independent contribution of skin temperature on heat-related changes in physical performance for people with MS.

MS patients’ current medications and lapse in time since optic neuritis diagnosis or recent exacerbation in visual symptoms unrelated to heat stress were not recorded as part of the study. Participant eligibility was determined by examination of patient medical records to ensure all MS patients were not taking steroids and had no exacerbation of visual symptoms unrelated to heat stress within the previous three months. Furthermore, the types of visual symptoms experienced by each MS participant during heating were not recorded.

### Impact

Many MS organisations, including MS Australia and the National MS Society, recommend cooling areas like the neck, wrists, or ankles to reduce heat-related performance declines in MS. Our inconclusive data suggest these recommendations may still be optimal if a large area of skin is cooled to more meaningfully reduce WBTS. The present study indicates that only slightly reducing WBTS alone with localised skin cooling does not mitigate heat sensitivity in people with MS during moderate rises in core temperature. Therefore, cooling a significant area of skin for a meaningful reduction in WBTS or lowering axonal temperature in the CNS achieved through limiting the rise in core temperature are likely the most effective remedies. Indeed, cold water immersion (16–17 ℃) to reduce core temperature by ~ 0.7 ℃ in people with MS before exercise was necessary to mitigate heat-related post-exercise fatigue and deterioration in functional performance relative to no pre-cooling (White et al. 2000). Similarly, many MS symptoms experienced at rest can be alleviated with cold water or cold air immersion that reduce core temperature by ~ 0.6 ℃ (Watson 1959).

## Conclusions

A marginally lower WBTS with lower back skin cooling did not mitigate heat-related decrements in visual performance in people with MS, unilateral optic neuritis and heat-sensitive visual symptoms. The limited perceptual difference achieved suggests the localised skin cooling was insufficient to meaningfully isolate the effects of skin temperature from core temperature. Hence, we were unable to properly test our hypothesis that reducing WBTS through localised skin cooling would mitigate visual performance decrements independently of a raised core temperature. Future studies should aim to test this hypothesis using a larger sample and a similar experimental design, but with a more effective intervention capable of meaningfully reducing WBTS while maintaining an elevated core temperature.

## Data Availability

The data that support the findings of this study are available from the corresponding author upon reasonable request.
